# The GMAP210 homologue SQL 1 modulates intraflagellar transport in *C. elegans*

**DOI:** 10.1186/2046-2530-1-S1-P40

**Published:** 2012-11-16

**Authors:** S Rademakers, J Broekhuis, M Dekkers, J Burghoorn, G Jansen

**Affiliations:** 1 Erasmus Medical Center Rotterdam, the Netherlands; 2 Biozentrum, University Basel, Switzerland

## 

The development and function of cilia require a specialised transport process, called Intraflagellar Transport (IFT). In amphid cilia of *C. elegans* this process uses two kinesins, kinesin II and OSM-3, which are loaded with complex A and B particle proteins and cargo molecules. We have previously shown that expression of a dominant active G-protein (GPA-3QL) in amphid channel neurons affects the coordination of OSM-3 and kinesin-II and results in shorter cilia. We performed a genetic screen to identify mutants that suppress the gpa-3QL cilia length defect and identified sql-1 (supressor of gpa-3QL), which encodes the homologue of the mammalian Golgi protein GMAP210. GMAP210 has been shown to play a role in vesicular transport from the Golgi apparatus to the cilium. SQL-1 is ubiquitously expressed in C. elegans and localizes to the Golgi. *sql-1 loss of function* (*lf*) mutants show wildtype length cilia, while animals overexpressing SQL-1 have longer cilia. Speed measurements in *sql-1*(*lf*) animals showed that OSM-3 moves faster and kinesin II moves slower, suggesting that the two kinesins are partially uncoupled. Complex A and B proteins move at the same speed as OSM-3, suggesting that IFT is predominantly mediated by OSM-3 kinesin. Interestingly, in the *gpa-3QL; sql-1*(*lf*) double mutants the speed of OSM-3 is decreased. We hypothesize that SQL-1 plays a role in routing or post translational modifications of proteins that are required in the cilium for proper IFT.

